# Quantitative Proteomics and Differential Protein Abundance Analysis after the Depletion of PEX3 from Human Cells Identifies Additional Aspects of Protein Targeting to the ER

**DOI:** 10.3390/ijms222313028

**Published:** 2021-12-01

**Authors:** Richard Zimmermann, Sven Lang, Monika Lerner, Friedrich Förster, Duy Nguyen, Volkhard Helms, Bianca Schrul

**Affiliations:** 1 Medical Biochemistry and Molecular Biology, Saarland University, 66421 Homburg, Germany; sven.lang@uni-saarland.de (S.L.); monika.lerner@uks.eu (M.L.); 2 Bijvoet Center for Biomolecular Research, Utrecht University, 3584 CH Utrecht, The Netherlands; f.g.forster@uu.nl; 3 Center for Bioinformatics, Saarland Informatics Campus, Saarland University, 66041 Saarbrücken, Germany; duy.nguyen@dkfz-heidelberg.de (D.N.); volkhard.helms@bioinformatik.uni-saarland.de (V.H.)

**Keywords:** endoplasmic reticulum, lipid droplets, peroxisomes, PEX3, protein targeting, membrane protein insertion, protein translocation, label-free quantitative mass spectrometry, differential protein abundance analysis, Zellweger syndrome

## Abstract

Protein import into the endoplasmic reticulum (ER) is the first step in the biogenesis of around 10,000 different soluble and membrane proteins in humans. It involves the co- or post-translational targeting of precursor polypeptides to the ER, and their subsequent membrane insertion or translocation. So far, three pathways for the ER targeting of precursor polypeptides and four pathways for the ER targeting of mRNAs have been described. Typically, these pathways deliver their substrates to the Sec61 polypeptide-conducting channel in the ER membrane. Next, the precursor polypeptides are inserted into the ER membrane or translocated into the ER lumen, which may involve auxiliary translocation components, such as the TRAP and Sec62/Sec63 complexes, or auxiliary membrane protein insertases, such as EMC and the TMCO1 complex. Recently, the PEX19/PEX3-dependent pathway, which has a well-known function in targeting and inserting various peroxisomal membrane proteins into pre-existent peroxisomal membranes, was also found to act in the targeting and, putatively, insertion of monotopic hairpin proteins into the ER. These either remain in the ER as resident ER membrane proteins, or are pinched off from the ER as components of new lipid droplets. Therefore, the question arose as to whether this pathway may play a more general role in ER protein targeting, i.e., whether it represents a fourth pathway for the ER targeting of precursor polypeptides. Thus, we addressed the client spectrum of the PEX19/PEX3-dependent pathway in both PEX3-depleted HeLa cells and PEX3-deficient Zellweger patient fibroblasts by an established approach which involved the label-free quantitative mass spectrometry of the total proteome of depleted or deficient cells, as well as differential protein abundance analysis. The negatively affected proteins included twelve peroxisomal proteins and two hairpin proteins of the ER, thus confirming two previously identified classes of putative PEX19/PEX3 clients in human cells. Interestingly, fourteen collagen-related proteins with signal peptides or N-terminal transmembrane helices belonging to the secretory pathway were also negatively affected by PEX3 deficiency, which may suggest compromised collagen biogenesis as a hitherto-unknown contributor to organ failures in the respective Zellweger patients.

## 1. Introduction

Analogously to the division of the human body into several organs, the nucleated human cell is divided into various different compartments, the cell organelles, which are surrounded—and thereby separated from the cytosol—by biological membranes. However, the vast majority of the approximately 30,000 types of the polypeptides—along with their isoforms—of a human cell are synthesized in the cytosol. Therefore, the proteins of the different organelles have to be sorted to the correct organelles and, subsequently, inserted into or translocated across the organellar membrane(s). The protein import into the endoplasmic reticulum (ER) is the first step in the biogenesis of about 10,000 different soluble and membrane proteins of human cells [1,2,3,4]. These were found to fulfill their functions in the membrane or lumen of the ER, along with the nuclear envelope, in one of the organelles belonging to the pathways of endo- and exocytosis (i.e., ERGIC, Golgi apparatus, endosome, lysosome, transport vesicles), or at the cell surface as plasma membrane- or secretory-proteins. Excluding resident proteins of the ER, most of the correctly folded and assembled proteins are delivered from the ER to their functional location by vesicular transport, which involves vesicles budding off from subdomains of the tubular ER, which are termed “exit sites” (ERES) [5,6]. In recent years, however, an increasing number of proteins destined to lipid droplets (LDs), peroxisomes or mitochondria were observed to be targeted to the ER as well, prior to their integration into budding LDs or peroxisomes, or prior to their delivery to mitochondria via the recently-identified ER–SURF pathway [7,8,9,10,11]. LDs and peroxisomes are ER-derived organelles, and their biogenesis occurs in specialized subdomains of the tubular ER [8,10].

**Figure 1:**
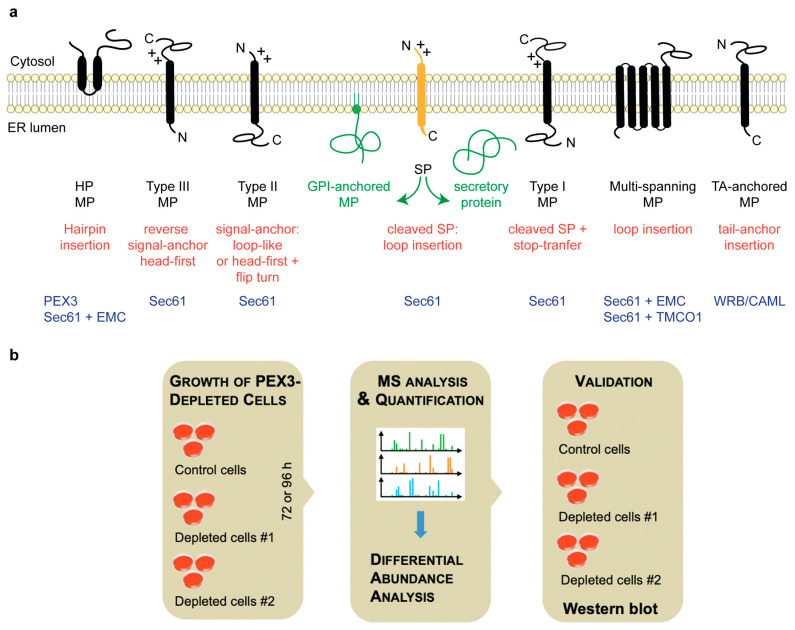
Types of ER membrane proteins and our experimental strategy to address their biogenesis. (**a**) The cartoon depicts a signal peptide (SP) (in yellow) and six types of ER membrane proteins (MP) (in black), together with their membrane protein type and the mechanism of membrane insertion (both indicated below the cartoon). Cleavable SPs (in yellow) can facilitate the ER import of secretory proteins (in green), glycosylphosphatidylinositol (GPI)-anchored membrane proteins (in green), and several types of membrane proteins, including single-spanning type I membrane proteins. Positively charged amino acid residues (+) play an important role in membrane protein and SP orientation, i.e., they typically follow the positive inside rule [14]. Amino-terminal transmembrane helices (TMHs) can serve as signal-anchor sequences to facilitate the membrane insertion of type II, type III, and many multi-spanning membrane proteins. In the case of membrane proteins with amino-terminal TMHs, membrane insertion typically involves the same components and mechanisms, which deliver secretory proteins (in green) and GPI-anchored membrane proteins (in green) to the ER lumen. The central component here is the Sec61 complex. In some cases, however, auxiliary membrane protein insertases, such as EMC or TMCO1 complex, play a role. These can also operate as stand-alone membrane protein insertases, an activity that they have in common with the WRB/CAML complex [4]. Hairpin (HP) proteins have a monotopic topology with N- and C-termini facing the cytosol, and some of them require PEX3 for membrane targeting. C, carboxy-terminus; N, amino-terminus. (**b**) The experimental strategy was as follows: siRNA-mediated gene silencing using two different siRNAs for each target and one non-targeting (control) siRNA, respectively, with three replicates for each siRNA for 96 h, followed by the label-free quantitative analysis of the total cellular proteome, and then differential protein abundance analysis to identify negatively affected proteins (i.e., putative clients of the target) and positively affected proteins (i.e., putative compensatory mechanisms), and finally validation by Western blot. In addition, PEX3-deficient Zellweger patient cells were analyzed in triplicates.

Protein import into the ER involves ER membrane targeting as the initial step, and the insertion of nascent or fully-synthesized membrane proteins into—or the translocation of soluble precursor polypeptides across—the ER membrane as the second step [1,2,3,4]. Typically, both processes depend on N-terminal signal peptides (SPs) or transmembrane helices (TMHs) in the precursor polypeptides that serve as signals [4,12,13,14]. Generally, the Sec61 complex of the ER membrane represents the entry point for most of these precursor polypeptides into the organelle (Table 1) [1,2,3,4]. A variety of proteins rely on the Sec61 complex for their integration into the ER lumen or their translocation across the ER membrane (Figure 1a). These include SP-containing soluble or GPI-anchored proteins, bitopic type I (C-in N-out) and type II (N-in C-out) proteins, and multispanning membrane proteins. Sec61 is also implicated in the membrane insertion of proteins without N-terminal SPs, such as type III (C-in N-out) [4] and C-tail-anchored (TA) (N-in C-out) membrane proteins [15,16,17], and even monotopic hairpin proteins (C-in N-in) [18,19]. In these cases, however, the role of Sec61 is less clear, and auxiliary factors such as the TRAP complex, the Sec62/Sec63 complex, or insertases such as the ER membrane protein complex (EMC) or the TMCO1 complex are required in addition [4]. Especially the membrane insertion of TA-proteins predominantly relies on the insertase WRB/CAML [15,16,17]. In conclusion, several co- and post-translational protein targeting pathways merge at the Sec61 complex in the ER membrane, including the co-translational SRP/SR-pathway and the post-translational SRP-independent or SND pathways [2,4,20,21,22,23,24,25,26].

In addition, there is the targeting of mRNAs to the ER membrane, which involves mRNA receptors (such as KTN1), or receptors for ribosome nascent chain complexes with nascent chains, which are not yet long enough to be able to interact with SRP (such as RRBP1) [27,28,29,30,31,32,33]. However, one general lesson from the analysis of all these pathways is that they are not strictly separated from each other, and there are at least some precursor polypeptides which can be targeted to the ER by more than one pathway (such as some small presecretory proteins and some tail-anchored membrane proteins) [23,25,26]. Thus, the targeting pathways have overlapping substrate specificities, and can substitute for each other, at least to a certain extent. The characterization of all of these pathways and mechanisms is also of medical importance, as several of the components are linked to human hereditary or tumor diseases, or are hijacked by viral or bacterial agents [34].

Recent work identified the PEX19/PEX3-dependent pathway as a fourth pathway for the ER targeting of precursor polypeptides [35,36]. PEX3 (also termed peroxisomal biogenesis factor 3 or Peroxin-3) was first identified in yeast, and is a membrane protein with an N-terminal transmembrane domain and a large C-terminal domain, which faces the cytosol both in yeast and in humans [37,38,39,40,41]. Originally, it was characterized as a peroxisomal membrane protein, which cooperates with the cytosolic protein PEX19 in the targeting of peroxisomal membrane proteins to pre-existent peroxisomes and in the facilitation of their membrane insertion [38,40]. However, PEX3 is also present in discrete subdomains of ER membranes, and is involved in the targeting of an unknown number of precursor proteins to ER membranes, and possibly in their membrane insertion [35,36]. So far, these precursor proteins include membrane proteins, which either remain in the ER (the two-hairpin or reticulon-domain containing proteins ARL6IP1, RTN3A, and RTN4C) [35] or are pinched off in LDs (such as the hairpin protein UBXD8) [36]. At the ER, PEX3 cooperates with the farnesylated variant of PEX19 [36,39]. Thus, the farnesylation of PEX19 is most likely decisive in delivering precursor polypeptides to either pre-existent peroxisomes or the ER [7,8]. These observations raised the question of whether this pathway may play a more general role in ER protein targeting. Defects in the human PEX3 gene are linked to a particularly devastating form of Zellweger syndrome, which belongs to the peroxisome biogenesis disorders, and is also termed “cerebro-hepato-renal syndrome” to indicate the most important affected organs [41,42,43,44]. Infants with the disease typically die within their first year of life because of the complete absence of peroxisomes in all of the cells of the body.

Here, we address the client spectrum of PEX3 in ER protein targeting in human cells and, simultaneously, the question of whether the PEX19/PEX3-dependent pathway to the ER can also target precursor polypeptides to the Sec61 complex. The approach involves transiently PEX3-depleted HeLa cells or chronically PEX3-deficient Zellweger patient fibroblasts in combination with differential proteomic analysis by label-free quantitative mass spectrometry (MS) and differential protein abundance analysis (Figure 1b). Thus, we report on negatively and positively affected proteins after the partial depletion of PEX3 in HeLa cells and in PEX3-deficient Zellweger patient fibroblasts.

## 2. Results

### 2.1. Quantitative Proteomic Analysis of HeLa Cells after the Transient and Partial Depletion of PEX3 by siRNA

Our approach to the characterization of the client spectrum of PEX3 in ER protein targeting involves the gene silencing of the putative receptor PEX3 in HeLa Kyoto cells with two different targeting siRNAs in parallel to a non-targeting or control siRNA, and differential proteomic analysis by label-free quantitative MS analysis and differential protein abundance analysis (Figure 1b). This protocol was developed and previously used to characterize the client spectrum and client SP features of ER protein translocation components, including Sec61 complex (as a proof on concept), TRAP complex, Sec62/Sec63 complex, TRAM1-protein, ERj1, BiP, and the mRNA targeting components KTN1 and RRBP1 [33,46,47,48]. The approach is based on the assumption that polypeptide precursors, which have to be imported into the ER, are degraded by the proteasome in the cytosol upon interference with their ER targeting or translocation because their SPs or TMHs are not easily compatible with the aqueous character of the cytosol. Therefore, their cellular levels are decreased compared to those of the control cells, and this change is detected by quantitative MS and subsequent differential protein abundance analysis [46]. Typically, the decrease was observed to be accompanied by an increase of ubiquitin-conjugating enzymes [33,46,47,48]. Furthermore, a simultaneous increase in other ER import components was detected, which is consistent with the overlap of pathways and, additionally, may indicate a genetic interaction between different pathways and cellular compensation.

Here, we applied the established experimental strategy to identify precursor polypeptides that may depend on PEX3-dependent targeting to the ER [46]. They were expected among the negatively affected proteins in the label-free quantitative MS and subsequent differential protein abundance analysis. HeLa cells were treated in triplicates with two different *PEX3*-targeting siRNAs (*PEX3* #1 siRNA, *PEX3* #2 siRNA) in parallel to a non-targeting (control) siRNA for 96 h. Each MS experiment provided proteome-wide abundance data as LFQ intensities for three sample groups: one control (non-targeting siRNA treated) and two stimuli (down-regulation by two different targeting siRNAs directed against the same gene), which each having three data points (Figure 1b). In order to identify which proteins were affected by knock-down in siRNA-treated cells relative to the non-targeting (control) siRNA treated sample, we log2-transformed the ratio between the siRNA and control siRNA samples, and performed two separate unpaired *t*-tests for each siRNA against the control siRNA sample according to [46]. The *p* values obtained by the unpaired *t*-tests were corrected for multiple testing using a permutation-based false discovery rate (FDR) test. The proteins with an FDR-adjusted *p* value of below 5% were considered significantly affected by the knock-down of the targeted protein. 

After PEX3 depletion, 6488 different proteins were quantitatively detected by MS in all of the samples (Figure S1, Table 2, Tables S1 and S2). The MS data were deposited to the ProteomeXchange Consortium via the PRIDE partner repository [49] with the dataset identifier PXD012005 (http://www.proteomexchange.org; last accessed on 2 May 2021). They included the expected representations of proteins with cleaved SP (7%), N-glycosylated proteins (9%), and membrane proteins (13%), which were comparable to the previously published Sec61 and TRAP experiments (Figure S1c, left small pies) [46]. Through the application of the established statistical analysis, we found that transient PEX3 depletion significantly affected the steady-state levels of 13 proteins: 13 negatively and none positively (with a permutation-based false discovery rate-adjusted *p* value < 0.05). As had to be expected, PEX3 itself was negatively affected (Figure S1a, volcano plots), which was confirmed by Western blot analysis (Figure S1b). The identified precursors included one protein with cleavable SP (the ER membrane protein AGPAT1), and two membrane proteins with TMH (not counting PEX3), i.e., the endo- and lysosomal membrane protein TMEM192 with four transmembrane domains, and the single-pass type II plasma membrane protein SGCD (Figure S1b, Table S2). Of these three negatively affected proteins, one was N-glycosylated (SGCD). There were no proteins with an annotated functional location in peroxisomes or LDs among the negatively affected proteins. There was no positively affected protein observed. These results raise the question of why PEX3 depletion from HeLa cells had hardly any effect on the cellular proteome. There are several possibilities. The simplest answer would be that the depletion efficiency of 85% may not have been high enough to cause the accumulation of precursor proteins. Another answer could be that PEX3 function in ER protein import in HeLa cells is not essential, i.e., it can be substituted by other proteins or pathways. Another possibility is that the accumulating precursors were not degraded but aggregated in the cytosol, or ended up in other organelles, where they were protected from degradation. These possibilities will we considered in further detail in the discussion.

### 2.2. Quantitative Proteomic Analysis of PEX3-Deficient Zellweger Patient Fibroblasts

**Figure 2:**
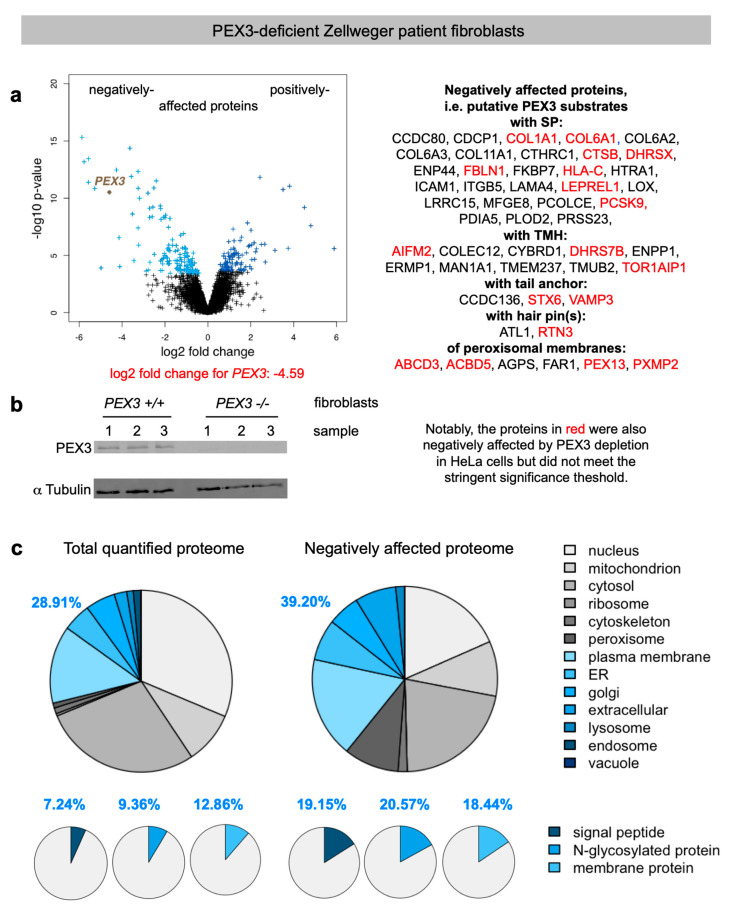
Volcano plots and Gene Ontology (GO) enrichment for PEX3-deficient Zellweger patient fibroblasts. (**a**) The differentially affected proteins were characterized by the mean difference of their intensities plotted against the respective permutation-based false discovery rate-adjusted *p*-values in the volcano plots; PEX3 is highlighted. In addition, the proteins, which were negatively affected by PEX3 deficiency are given in the right panel. (**b**) PEX3 deficiency was evaluated by Western blot. The molecular mass values are indicated in kilodaltons (KDa). Only the area of interest of the blot is shown; the original images are shown in the Supplementary Materials. (**c**) The classification of the putative PEX3 clients was based on GO enrichment factors where the results from the complete set of quantified proteins in the left panel are compared with the negatively affected proteome. The protein annotations of the SPs, membrane location, and N-glycosylation in humans were extracted from UniProtKB, and were used to determine the enrichment of the GO annotations among the negatively affected proteins.

In the course of our previous analysis of Sec62- and Sec63 clients, we moved from the respective siRNA-treated and incompletely depleted HeLa cells with low client numbers on to CRISPR/Cas9-treated and deficient HEK293 cells, and indeed, we identified many more clients [47]. Therefore, we sought to test whether a complete PEX3 knockout in cells leads to the depletion of putative PEX3 client proteins. Therefore, we subjected control fibroblasts and immortalized Zellweger patient fibroblasts with PEX3 deficiency [41], which had been grown in triplicates, to label-free quantitative proteomic analysis and differential protein abundance analysis, and analyzed the data for negatively affected proteins, i.e., potential PEX3 clients (Figure 2, Table 2, Tables S3–S5). The MS data were deposited to the ProteomeXchange Consortium via the PRIDE partner repository [49] with the dataset identifier PXD012005 (http://www.proteomexchange.org; last accessed on 2 May 2021).

We quantitatively identified a total of 6328 different proteins by MS, 141 of which were negatively affected by PEX3 deficiency in the patient fibroblasts versus the control fibroblasts. As had to be expected, PEX3 itself was negatively affected (Figure 2a, volcano plots), which was confirmed by Western blot (Figure 2b). Applying the established statistical analysis, we found that PEX3 deficiency significantly affected the steady-state levels of 238 proteins: 141 negatively and 97 positively (permutation-based false discovery rate-adjusted *p* value < 0.05). Of the negatively affected proteins, GO terms assigned 39.2% to organelles of the endocytic and exocytic pathways (Figure 2c, large pies), which corresponds to a 1.36-fold enrichment (Figure 2c, large pies, 39.2% divided by 28.91% = 1.36) and is below the average values of 1.46 and 1.94 observed after the depletion of the mRNA targeting components KTN1 (1.55) and RRBP1 (1.37), and the translocation components Sec61 (2.37) and TRAP (1.5), respectively (Table 2) [33,46]. In contrast to the PEX3-knock-down cells (Figure S1c), we also detected the enrichment of proteins with SP (2.65–fold), N-glycosylated proteins (2.2-fold), and membrane proteins (1.36–fold) (Figure 2c, small pies), which was lower compared to the Sec61 (6.51, 2.83, 2.51) and TRAP experiments (3.3, 2.7, 2.1), but higher compared to the KTN1 (1, 2.09, 1.76) and RRBP1 experiments (2.44, 2.12, 1.46) [33,46].

The negatively affected proteins of the secretory pathway included 27 proteins with cleavable SP (including six collagens or collagen-like proteins, three collagen-modifying proteins, three ER lumenal proteins—i.e., FKBP7, PCSK9, and PDIA5—the lysosomal cathepsin CTSB, eight secretory proteins, the ER membrane protein PLOD2, and the plasma membrane proteins ENPP4, HLA-C, ICAM1, ITGB5, and LRRC15 (Figure 2a).

Furthermore, 15 membrane proteins of the secretory pathway with TMH (not counting PEX3) were negatively affected, i.e., most notably the two ER-resident hairpin proteins ATL1 and RTN3; the three tail-anchored proteins CCDC136, STX6 and VAMP3; the ER membrane proteins DHRS7B, ERMP1, and TMUB2; the Golgi protein MAN1A1; the plasma membrane proteins AIFM2 (which has additional locations, see below), COLEC12, CYBRD1, ENPP1, and TMEM237; and the nuclear envelope protein TOR1AIP1 (Figure 2a, Table 3 and Table S4). Of these 42 negatively affected proteins, 29 were N-glycosylated proteins (23 with SP and 6 with TMH).

Interestingly, there were 14 precursors of mitochondrial proteins negatively affected by PEX3 deficiency (Table S4), a phenomenon previously observed after the depletion of RRBP1 from HeLa cells and attributed to their physiological trafficking from the ER to mitochondria via the newly identified ER-SURF pathway [11,33]. Among these negatively affected mitochondrial proteins were three outer membrane proteins (AIFM2, RHOT1, VAT1), one inner membrane protein (NDUFV3), and ten matrix proteins, two of which had a dual localization in the mitochondria and peroxisomes (ACAD11, SCP2). Alternatively, our observation of mitochondrial proteins among the negatively affected ones may be due to the findings that PEX19, at least, associates with peroxisomes and mitochondria, and, for example, facilitates the protein biogenesis of the tail-anchored membrane proteins Fis1 and Gem1 for both organelles [50].

The positively affected proteins in the PEX3-deficient fibroblasts included cell adhesion molecules (such as ITGA1, L1CAM, and NCAM1), signal transduction components of the plasma membrane (ANXA3, PKD2), mitochondrial membrane proteins of the inner membrane with functions in protein import into the mitochondria (DNAJC15) or protein quality control (OMA1), and the cytosolic chaperone HSPB6.

### 2.3. Negatively Affected Precursor Proteins in Zellweger Patient Fibroblasts Are Specific for PEX3- Deficiency, and Are Partially Affected in PEX3-Depleted HeLa Cells

**Figure 3:**
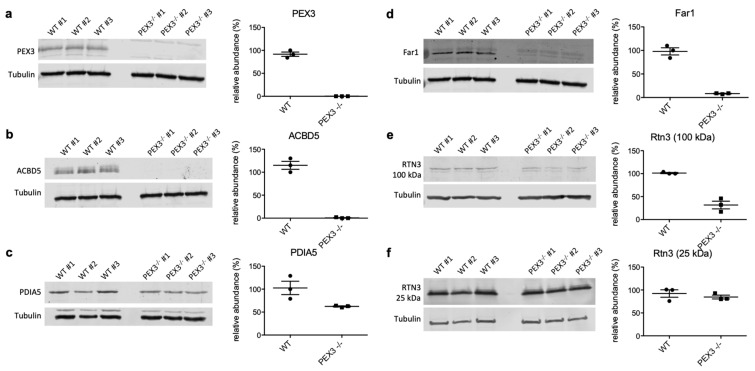
Validation of the PEX3 clients by Western blot analyses. (**a**–**f**) Three independent cell lysates from the control (WT) and PEX3-deficient fibroblasts (PEX3^−/−^), respectively, were analyzed by Westen blotting using antibodies as indicated. Left panels: Relevant sections of the representative Western blots are shown; tubulin served as a loading control. We note that the full scans of all of the blots are shown in the supplement. Right panels: The scatter plots indicate the relative protein abundances in the control and PEX3-deficient fibroblasts, as derived from quantitative Western blots, as shown in the left panels. The signals were quantified by densitometry, and the relative abundances were calculated as the ratio of the signal of interest to the corresponding tubulin signal in the same lane, and were normalized against one control sample. The mean values with SEM from three independent lysates per cell line are indicated, as well as the individual data points for each replicate.

In order to validate the proteomic data on putative PEX3-substrates, we conducted independent Western blot experiments with the PEX3-deficient Zellweger and control fibroblasts for the SP-containing candidate PDIA5, the hairpin protein RTN3, the dual topology LD/peroxisome protein Far1, and the peroxisomal protein ACBD5 (Figure 3). All of these proteins were depleted in PEX3-deficient fibroblasts, fully confirming the proteomic analysis and verifying them as putative PEX3 clients. Interestingly, for RTN3, we observed that specifically the 100 kDa isoform was depleted in the PEX3-deficient cells, while the 25 kDa isoform remained largely unaltered (Figure 3, e versus f).

For PEX3-depleted HeLa cells, we observed that 17 of the 48 negative hits were not quantified, and that 18 out of 31 putative PEX3 clients in Zellweger patient fibroblasts (i.e., 58%) were also negatively affected by PEX3 depletion in HeLa cells (including two out of three tail-anchored proteins, one out of two hairpin proteins, and four out of six peroxisomal proteins), but did not meet the stringent significance threshold (Table S6 and proteins indicated in red in Figure 2). Notably, the PEX3 depletion in the HeLa cells was not as efficient as it was in the fibroblasts (log2-fold change: -3.5 versus -4.6) (Figure 2 and Figure S1). Hence, it is understandable that the levels of the putative PEX3 clients are less perturbed in HeLa cells than in fibroblasts. Still, the majority of them (18 versus 13) are perturbed in the same negative direction as they are in fibroblasts (Table S1).

We consider these putative clients of PEX3 for organelles of the secretory pathway to be specific for additional reasons. First, the negatively affected proteins included, as expected, twelve precursors of proteins with a functional location in peroxisomes (not counting PEX3), including six peroxisomal membrane proteins (Figure 2a) (Table 3). The peroxisomal membrane proteins were ABCD3, ACBD5, AGPS, FAR1, PEX13, and PXMP2. Notably, the tail-anchored membrane protein FAR1 exhibits a dual topology, and can locate to peroxisomes as well as to LDs [51]. Second, with two hairpin proteins, ATL1 and RTN3, among the negatively affected proteins, we also confirmed a second class of already-known PEX3 clients in human cells under physiological conditions (Table 3). Third, only one of the negatively affected peroxisomal proteins, PEX13, had previously been observed for TRAP-deficient fibroblasts from patients who suffer from congenital disorders of glycosylation (CDG) and are either *SSR3-* (coding for TRAPγ) or *SSR4* (TRAPδ)-deficient [46]. Furthermore, there was no overlap in the positively affected proteins that accumulate in either CDG or Zellweger patient fibroblasts.

As outlined in Table 2, and for comparison with the PEX3-deficient Zellweger patient fibroblasts, 5919 different proteins were previously quantified for CDG patient fibroblasts, 279 of which were negatively affected by TRAP absence, and 39 of which were positively affected. In total, 100 of the negatively affected proteins were assigned to the secretory pathway, including 34 precursor polypeptides with SP and 41 with TMH (including the subunits of the heterotetrameric TRAP complex) [46]. A total of 47 of the negatively affected proteins were N-glycoproteins (30 with SP and 17 with TMH). The peroxisomal membrane protein PEX13 was among the negatively affected proteins, which was also negatively affected in PEX3-deficient Zellweger patient fibroblasts (Table 2).

## 3. Discussion

Here, we addressed the question of which precursor polypeptides employ the PEX3-dependent pathway for the targeting of or insertion and translocation, respectively, into the ER of human cells. First, we employed our previously established approach of the siRNA-mediated depletion of PEX3 in HeLa cells, the label-free quantitative MS of the total cellular proteome, and differential protein abundance analysis. Next, we quantified the negatively and positively affected proteins under conditions of PEX3 deficiency in Zellweger patient fibroblasts.

On first sight, the result of the siRNA-mediated depletion of PEX3 in HeLa cells was not very informative (Figure S1). In addition to the depletion of PEX3, only three precursor polypeptides with SP or TMH were found among the negatively affected proteins and may be considered as PEX3 clients in ER protein targeting. Notably, the one with an SP, AGPAT1, is an important enzyme in lipid metabolism, and is required for the synthesis of phosphatidic acid and triacylglycerides [52]. Therefore, it may affect LD biogenesis, and LD localization would not be unexpected. The proteins with a TMH are the endo- and lysosomal membrane protein TMEM192 with four transmembrane domains, and the single-spanning type II plasma membrane protein SGCD. There was no peroxisomal protein negatively affected by PEX3 depletion, which has to be interpreted in light of the facts that PEX19 and PEX3 are essential for peroxisome formation [37,38], and that PEX3 deficiency causes the complete absence of peroxisomes [42,43,44]. Together, these results raise the question of why PEX3 depletion from HeLa cells had hardly any effect on the cellular proteome. There are several possibilities. (i) The simplest answer would be that the depletion efficiency and its duration may not have been sufficient to cause a significant accumulation of precursor proteins. According to the MS data, the log2 fold change was −3.4942, i.e., the depletion efficiency was higher than 90%, which is consistent with the Western blot analysis (Figure S1b). This residual amount of PEX3, however, may have been sufficient for physiological functions, and could explain the absence of an effect on peroxisomal proteins. (ii) Another answer may be that the PEX3 function in ER protein import in HeLa cells is not essential, i.e., it may also be provided by other proteins or pathways. Indeed, it was shown in cell-free ER protein import studies that certain peroxisomal membrane proteins can be targeted to the mammalian ER by SRP or TRC40 (including PEX3) [29,35,53,54,55]. Furthermore, some collagens, as well as some hairpin membrane proteins, were previously observed as RRBP1 clients (ATL2, ATL3, COL1A1, COL1A2, COL4A2) and SRP clients (ARL6IP1, RTN3), respectively, in cell biological or proteomic experiments [30,33,35]. (iii) Another possibility is that some accumulating precursors were not degraded, and either stayed soluble in the cytosol (as is known for catalase), aggregated, or ended up in other organelles (as is known for PEX14) [41], where they were protected from degradation. Indeed, we have previously observed the mis-targeting of certain precursors of secretory proteins into mitochondria in the absence of Sec61 function in HeLa cells [56]. Furthermore, the known PEX3 client, UBXD8, accumulates in mitochondria when PEX3 function is compromised [36]. (iv) Last but not least, all three possibilities may have contributed to the result we obtained in HeLa cells upon siRNA-mediated knockdown; we consider this the most likely explanation.

**Figure 4:**
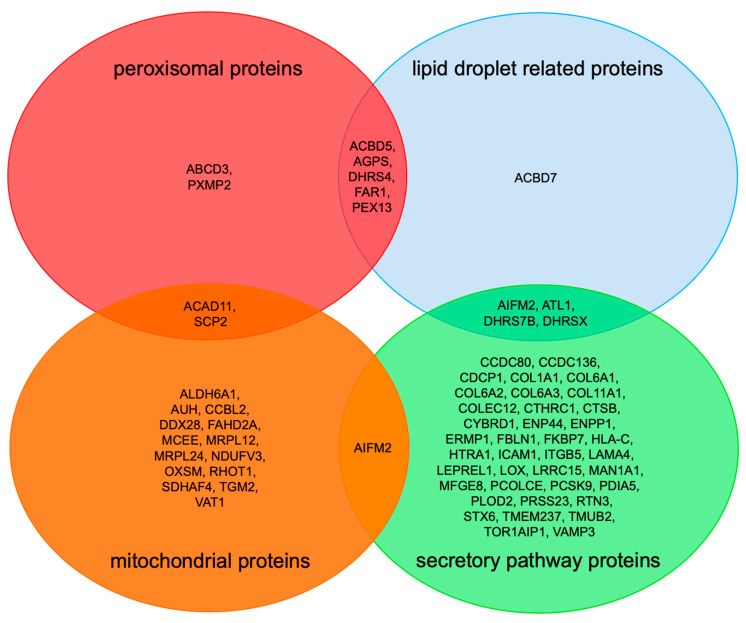
Venn diagram for negatively affected proteins in PEX3-deficient Zellweger patient fibroblasts. We note that Table 3 and Table S4 served as the basis for this compilation.

Under conditions of PEX3 deficiency in Zellweger patient fibroblasts, the results were more informative (Figure 2). First, the negatively affected proteins included twelve precursors of proteins with a functional location on or in peroxisomes, including six peroxisomal membrane proteins (Figure 4, Table 3). This was expected, and demonstrated once more the feasibility of the approach. However, this does not mean that all of these precursors of peroxisomal proteins are targeted to the ER. Rather, at least for the peroxisomal matrix proteins, it must be due to the complete absence of peroxisomes from the patient fibroblasts with PEX3 deficiency [42,43,44]. Likewise, we cannot rule out that the depletion of some precursors could derive from transcriptional effects. However, we consider this unlikely, as previous transcriptomics studies revealed that solely the absence of peroxisomes does not generally result in global mRNA expression changes [57].

Second, the identified precursors included the two ER-resident hairpin proteins ATL1 (with one hairpin) and RTN3 (with two hairpins), which is consistent with the previous finding that PEX3 is involved in the ER targeting of hairpin proteins [35,36]. It remains to be tested whether PEX3, possibly in cooperation with PEX16 [41], also facilitates the membrane insertion of these hairpin proteins, and whether additional membrane protein insertases—such as Sec61, EMC, TMCO1, and WRB/CAML—contribute to membrane insertion. Likewise, it is also conceivable that the members from the DHRS- and ACBD-protein families (ACBD5, DHRS4, DHRS7b, and DHRSX; Figure 2, Table 3) are first inserted into the ER membrane by PEX3 and additional insertion components of the ER. This will also have to be addressed in future research.

Furthermore, 14 α-helical membrane proteins, including four tail-anchored membrane proteins (CCDC136, FAR1, STX6 and VAMP3) and ten others (not counting PEX3)—i.e., DHRS7B (type II), ERMP1 (multi-pass), TMUB2 (multi-pass), MAN1A1 (type II), AIFM2, COLEC12 (type II), CYBRD1 (multi-pass), ENPP1 (type II), TOR1AIP1 (single-pass), and TMEM237 (multi-pass)—were negatively affected by PEX3 absence, and therefore can be considered as potential PEX3 clients in ER protein targeting (Table 3). Importantly, the annotated membrane topology of these potential clients should be considered with care, as they may be, in certain cases, incomplete. For example, it was recently shown that the tail-anchored membrane protein FAR1 exhibits a dual topology, including a monotopic hairpin topology, and can therefore locate to peroxisomes as well as LDs [51]. Because ERMP1, STX6, and TOR1AIP1 were previously found to depend on Sec61 for their membrane insertion in HeLa cells, it is tempting to speculate that PEX3 is able to target precursors to the Sec61 complex.

In addition, the identified precursors included 27 proteins with cleavable SP, including five collagens and one collagen-like protein, four collagen-modifying proteins LE–REL1, three ER lumenal proteins, and the four plasma membrane proteins with a connection to the extracellular matrix or neighboring cells, i.e., CDCP1, ICAM1, ITGB5, LRRC15. Thus, between 14 and 17 of the total of 43 secretory pathway precursor proteins are collagens or related to collagens, which is a significant enrichment of 33–40%. This raises the question of how PEX3 could be involved in the targeting of these precursors, which are expected to involve a cotranslational targeting pathway to the Sec61 complex. We hypothesize that, in these particular cases, PEX3 may act in concert with mRNA targeting pathways, which would be consistent with the observation that RRBP1-mediated targeting pathways were found to be involved in the biogenesis of these proteins [30,33]. However, their degradation in the absence of PEX3 may have an alternative explanation. We speculate that the combination of farnesylated PEX19 and ER-membrane-resident PEX3 targets the membrane-shaping and hairpin(s)-containing membrane proteins (such as Atlastins, Reticulons and Spastin) to the PEX3-rich ER subdomain, and that this enrichment of hairpin proteins creates an environment which attracts collagens as well as some of their modifying enzymes and future interaction partners [6,58,59,60,61]. As is consistent with this scenario, the key player of the formation of large cargo secretory vesicles at ER exit sites, the membrane protein TANGO1, has two transmembrane domains, one of which is supposed to form a hairpin in the inner leaflet of the ER membrane [59,60]. Either way, a common ER subdomain may be conducive to the budding of both peroxisomal precursor vesicles and large cargo secretory vesicles, as well as the formation of LDs. Indeed, very recent evidence suggests that membrane bridges between ER exit sites and LDs allow the protein partitioning of hairpin proteins from the ER to LDs (doi: https://doi.org/10.1101/2021.09.14.460330; accessed on 25 November 2021). In addition, this observation raises the question of whether defects in collagen biogenesis contribute to the devastating effects of PEX3 deficiency in Zellweger patients, which clearly warrants further work.

Furthermore, negatively affected proteins in PEX3-depleted or -deficient human cells need to be discussed with respect to LD biogenesis from a functional point of view (Figure 3, Table 3). DHRS- and ACBD-family proteins, which were negatively affected in PEX3-deficient fibroblasts (Table 3) and the dehydrogenase/reductase SDR family members 4, X and 7B (DHRS4, DHRSX, DHRS7B) play important roles in retinol biosynthesis, and may also localize to LDs [62]. The latter was suggested for DHRSX, and was actually shown for the family members DHRS3 and DHRS7B [62] (doi: https://doi.org/10.1101/2021.09.14.460330). On the other hand, acyl-CoA-binding domain-containing proteins 5 and 7 (peroxisomal ACBD5, cytosolic ACBD7) are involved in organelle contacts and, therefore, may be important for lipid metabolism and/or organelle budding from the ER. These aspects, too, warrant further studies.

Taken together, our study revealed a putative client spectrum for PEX3-mediated protein targeting to the ER, including several unexpected protein classes, such as secreted collagens. Importantly, several PEX3 client candidates are involved in lipid metabolic pathways or membrane-shaping mechanisms, which affect the ER-derived organelle biogenesis of peroxisomes and/or LDs. As observed for other ER targeting pathways, PEX3 also likely shares some of its putative clients with other ER targeting components, including the Sec61 complex.

## 4. Materials and Methods

### 4.1. Cell Growth and Analysis

HeLa Kyoto cells [36] were cultivated at 37 °C in a humidified environment with 5% CO_2_, in DMEM with 10% fetal bovine serum (FBS; Sigma-Aldrich, Taufkirchen, Germany). The cell growth and viability were monitored using the Countess^®^ Automated Cell Counter (Invitrogen, Thermo Fisher Scientific, Darmstadt, Germany), following the manufacturer’s instructions.

For gene silencing, 4 × 10^5^ HeLa cells were seeded per 6-cm culture plate, followed by incubation under normal culture conditions. Next, the cells were transfected with either PEX3-targeting Silencer Select pre-designed siRNA (Life Technologies, Darmstadt, Germany, IDs s16154 and s16156) or with a scrambled siRNA control (Life Technologies ID 4390843) at a final concentration of 3.3 nM using Lipofectamine 2000 (Life Technologies), following the manufacturer’s instructions. After 48 h, the cells were transfected a second time and grown for an additional 48 h. Thus, silencing was performed for a total of 96 h using two different siRNAs. We note that the cell viability was not affected by PEX3 depletion for 96h, i.e., the viability values were virtually identical for the two PEX3-targeting siRNAs (95.0% for RNA#1 and 96.0% for RNA#2, *n* = 3) versus the scrambled siRNA control (95.7%).

The silencing efficiencies were evaluated by Western blot analysis using PEX3-specific antibodies [63] (1:1000 dilution), which were kindly donated by Gabriele Dodt (University of Tübingen, Tübingen, Germany) and anti-tubulin antibodies (T6199, Sigma-Aldrich; 1:10,000 dilution). Donkey-derived, Cy3- and Alexa488-conjugated secondary antibodies (715-165-151, 711-545-152, Jackson Immunoresearch, Cambridgeshire, UK) were detected using the Typhoon-Trio imaging system combined with Image Quant TL software 7.0 (GE Healthcare, Freiburg, Germany).

Immortalized PEX3-deficient fibroblasts and control fibroblasts were obtained from Gabriele Dodt (University of Tübingen, Tübingen, Germany), and were previously characterized [41,64]. They were cultivated at 37 °C in a humidified environment with 5% CO_2_, in DMEM/GlutaMAX with 10% fetal bovine serum (FBS; Sigma-Aldrich) for 72 h.

### 4.2. Label-Free Quantitative Proteomic Analysis

After growth for 96 h, 1 × 10^6^ cells (corresponding to roughly 0.2 mg protein) were harvested, washed twice in PBS, and lysed in a buffer containing 6 M GnHCl, 20 mM tris(2-carboxyethyl)phosphine (TCEP; Pierce^TM^, Thermo Fisher Scientific, Darmstadt, Germany), and 40 mM 2-chloroacetamide (CAA; Sigma-Aldrich) in 100 mM Tris, at pH 8.0 [33,46,47,48]. The lysate was heated to 95 °C for 2 min, and then sonicated in a Bioruptor sonicator (Diagenode, Seraing, Belgium) at the maximum power setting for 10 cycles of 30 s each. For a 10% aliquot of the sample, the entire process of heating and sonication was repeated once, and then the sample was diluted 10-fold with digestion buffer (25 mM Tris, pH 8, 10% acetonitrile). The protein extracts were digested for 4 h with Lysyl endoproteinase Lys-C (Wako Bioproducts, Fujifilm, Neuss, Germany, enzyme to protein ratio: 1:50), followed by the addition of trypsin (Promega, Heidelberg, Germany) for overnight digestion (at an enzyme to protein ratio of 1:100). The next day, a booster digestion was performed for 4 h using an additional dose of trypsin (enzyme to protein ratio: 1:100). After the digestion, a 10% aliquot of peptides (corresponding to about 2 µg of peptides) were purified via SDB-RPS StageTips [65], eluted as one fraction, and loaded for MS analysis. Purified samples were loaded onto a 50-cm column (inner diameter: 75 microns; packed in-house with ReproSil-Pur C18-AQ 1.9-micron beads, Dr. Maisch HPLC GmbH, Ammerbuch, Germany) via the autosampler of the Thermo Easy-nLC 1000 (Thermo Fisher Scientific) at 60 °C. Using the nanoelectrospray interface, the eluting peptides were directly sprayed onto the benchtop Orbitrap mass spectrometer Q Exactive HF (Thermo Fisher Scientific) [66]. The peptides were loaded in buffer A (0.1% (*v*/*v*) formic acid) at 250 nL/min, and the percentage of buffer B was ramped to 30% over 180 min, followed by a ramp to 60% over 20 min, then 95% over the next 10 min, and maintained at 95% for another 5 min [33,47]. The mass spectrometer was operated in a data-dependent mode, with survey scans from 300 to 1700 m/z (resolution of 60,000 at m/z = 200). Up to 15 of the top precursors were selected and fragmented using higher energy collisional dissociation (HCD) with a normalized collision energy value of 28 [33,47]. The MS2 spectra were recorded at a resolution of 17,500 (at m/z = 200). The AGC targets for the MS and MS2 scans were set to 3E6 and 1E5, respectively, within a maximum injection time of 100 and 25 ms for the MS and MS2 scans, respectively. Dynamic exclusion was enabled in order to minimize the repeated sequencing of the same precursor ions, and was set to 30 s [33,47].

### 4.3. Data Analysis

The raw data were processed using the MaxQuant computational platform [67]. The peak list was searched against Human Uniprot databases, and the proteins were quantified across the samples using the label-free quantification algorithm in MaxQuant as the label-free quantification (LFQ) intensities [68]. We note that LFQ intensities do not reflect true copy numbers because they depend not only on the amounts of the peptides but also on their ionization efficiencies; thus, they only served to compare the abundances of the same protein in different samples [66,67,68,69,70,71]. Each MS experiment provided proteome-wide abundance data as LFQ intensities for three sample groups—one control (the non-targeting siRNA treated) and two stimuli (down-regulation by two different targeting siRNAs directed against the same gene)—with each having three data points. The missing data points were generated by imputation, whereby we distinguished two cases [46]. In order to identify which proteins were affected by PEX3 knock-down in siRNA-treated cells relative to the non-targeting (control) siRNA-treated sample, we log2-transformed the ratio between siRNA and the control siRNA samples, and performed two separate unpaired *t*-tests for each siRNA against the control siRNA sample [46]. The *p* values obtained by the unpaired *t*-tests were corrected for multiple testing using a permutation-based false discovery rate (FDR) test. The proteins with an FDR-adjusted *p* value of below 5% were considered to be significantly affected by the knockdown of the targeted protein. The results from the two unpaired *t*-tests were then intersected for further analysis, meaning that the abundance of all of the reported candidates was statistically significantly affected in both siRNA silencing experiments. For completely missing proteins lacking any valid data points, the imputed data points were randomly generated in the bottom tail of the whole proteomics distribution, following the strategy in the Perseus software (http://maxquant.net/perseus/; last accessed on 2 May 2021) [70]. For proteins with at least one valid MS data point, the missing data points were generated from the valid data points based on the local least squares (LLS) imputation method [71]. The validity of this approach was demonstrated [46]. Subsequent to the data imputation, gene-based quantile normalization was applied to homogenize the abundance distributions of each protein with respect to the statistical properties. All of the statistical analyses were performed using the R package of SAM (https://statweb.stanford.edu/~tibs/SAM/; last accessed on 2 May 2021) [72]. The protein annotations of the signal peptides, transmembrane regions, and N-glycosylation sites in humans and yeast were extracted from UniProtKB entries using custom scripts [46]. The enrichment of the functional Gene Ontology annotations (cellular components and biological processes) among the secondarily affected proteins was computed using the GOrilla package [73].

### 4.4. Validation of Putative PEX3 Substrates by Quantitative Western Blotting

Hits from the protemics analysis were validated by quantitative Western blot analyses of Triton-X100 cell lysates (1% Triton X-100, 50 mM Hepes pH 7.5, 150 mM NaCl, 10% glycerol, 1 mM EDTA, 1 mM PMSF, Complete EDTA-free protease inhibitors (Roche)) using the following antibodies: anti-PEX3 (gift from G. Dodt; 1:1000 dilution), anti-ACBD5 (HPA012145 Merck, Taufkirchen, Germany; 1:1000 dilution), anti-RTN3 (12055-2-AP Proteintech, Manchester, UK; 1:1000 dilution), anti-PDIA5 (15545-1 Proteintech; 1:1000 dilution), anti-Far1 (ATA-HPA017322 Biozol, Eching, Germany; 1:1250 dilution), and anti-tubulin (T6199, Sigma-Aldrich; 1:10,000 dilution), which served as a loading control for normalization. The secondary antibodies were purchased from Licor Biosciences, Bad Homburg, Germany (926-68020, 926-68021, 926-32211, 926-32210, all in 1:20,000 dilution). The signals were detected using the Odyssey Clx system from Licor Biosciences, and were quantified by densitometry using the Image Studio software (Licor Biosciences). The relative protein abundance was calculated as the ratio of the signal of interest to the corresponding tubulin signal in the same lane, and was normalized against one control sample. The visualization of the quantification data was performed using Graphpad Prism software.

## 5. Conclusions

Recent studies characterized the PEX19/PEX3 pathway, which is best known for its role in the biogenesis of peroxisomal membrane proteins both at the peroxisomal and the ER membrane, as being involved in the biogenesis of hairpin membrane proteins of the ER as well as LDs. Therefore, the question arose as to whether this pathway may play a more general role in ER protein targeting, i.e., whether it may represent a fourth pathway for the ER targeting of precursor polypeptides next to SRP, SND, and TRC/GET. We have started to address this question by a novel approach which involves the label-free quantitative mass spectrometry of the total proteome of depleted or deficient cells, along with differential protein abundance analysis. Thus, we addressed the client spectrum of the PEX19/PEX3-dependent pathway in both PEX3 targeting siRNA-treated HeLa cells and PEX3-deficient Zellweger patient fibroblasts. The negatively affected proteins included six peroxisomal membrane proteins and two hairpin proteins of the ER, thus confirming the two previously identified classes of putative PEX19/PEX3 clients for ER targeting in human cells, as well as the validity of the experimental approach. In addition, 14 membrane proteins (including four tail-anchored proteins) and 27 proteins with SP (including 14 collagens and collagen-related proteins) belonging to the secretory pathway were also negatively affected by PEX3 deficiency. The latter findings are consistent with the idea that PEX3 represents a fourth pathway for the targeting of precursor polypeptides to the Sec61 complex. Furthermore, it may suggest a hitherto unknown spatial—or at least physical—relationship between the ER subdomains that are involved in ER shaping and the budding of peroxisomal precursor vesicles, large cargo vesicles, and lipid droplets. In addition, these results may suggest compromised collagen biogenesis as a hitherto unknown contributor to organ failures in the respective Zellweger patients. All of these suggestions will have to be addressed in future research.

## Figures and Tables

**Table 1:** Protein transport components and associated proteins in HeLa cells and linked diseases.

Component/Subunit for ER Targeting	Abundance ^1^	Localization ^2^	Linked Diseases
#p34 (LRC59, LRRC59) ^3^	2480	ERM	
#p180 (RRBP1)	135	ERM	Hepatocellular Carcinoma, Colorectal Cancer
Kinectin 1 (KTN1)	263	ERM	
AEG-1 (LYRIC, MTDH)	575	ERM	
*#SRP* ^4^		C	
− SRP72	355		Aplasia, Myelodysplasia
− SRP68	197		
− SRP54	228		Neutropenia, Pancreas Insufficiency
− SRP19	33		
− SRP14	4295		
− SRP9	3436		
− 7SL RNA			
*SRP receptor*		ERM	
− SRα (docking protein)	249		
− SRβ	173		
Calmodulin	9428	C	
**for ER targeting and, possibly, for membrane integration**			
hSnd1	unknown		
*Snd receptor*		ERM	
− hSnd2 (TMEM208)	81		
− hSnd3 ^§^	49		
PEX19	80	C	Zellweger Syndrome
PEX3	103	ERM, PexM	Zellweger Syndrome
**for ER targeting plus membrane integration**			
*#Bag6 complex*		C	
− TRC35 (Get4)	171		
− Ubl4A	177		
− Bag6 (Bat3)	133		
SGTA	549	C	
TRC40 (Asna1, Get3)	381	C	
*TA receptor*		ERM	
− CAML (CAMLG, Get2)	5		
− WRB (CHD5, Get1)	4		Congenital Heart Disease
**for ER membrane integration**			
*ERM protein complex*		ERM	
− EMC1	124		
− EMC2	300		
− EMC3	270		
− EMC4	70		
− EMC5 (MMGT1)	35		
− EMC6 (TMEM93)	5		
− EMC7	247		
− EMC8	209		
− EMC9	1		
− EMC10	3		
#*TMCO1 complex* ^§^		ERM	Glaucoma, Cerebrofaciothoracic Dysplasia
− TMCO1	2013		
− Nicalin	99		
− TMEM147	21		
− CCDC47 (Calumin)	193		
− NOMO	267		
*PAT complex*		ERM	
− PAT10 (Asterix)			
− CCDC47 (Calumin)	193		
**for ER membrane integration plus translocation**			
*#Sec61 complex* ^§^		ERM	
− Sec61α1	139		Diabetes ^5^, CVID ^6^, TKD, Neutropenia
− Sec61β	456		PLD, Colorectal cancer
− Sec61γ	400		GBM, Hepatocellular carcinoma
#Sec62 (TLOC1)	26	ERM	Breast-, Prostate-, Cervix-, Lung-cancer
*ER Chaperones*			
− Sec63 (ERj2)	168	ERM	PLD, Colorectal cancer
− #ERj1 (DNAJC1)	8	ERM	
− BiP (Grp78, HSPA5)	8253	ERL	HUS
− Grp170 (HYOU1)	923	ERL	
− Sil1 (BAP)	149	ERL	MSS
#Calnexin_palmitoylated_	7278	ERM	
#TRAM1	26	ERM	
TRAM2	40	ERM	
*#TRAP complex*		ERM	
− TRAPα ((SSR1)	568		
− TRAPβ (SSR2)			
− TRAPγ (SSR3)	1701		CDG, Hepatocellular Carcinoma
− TRAPδ (SSR4)	3212		CDG
#RAMP4 (SERP1)		ERM	
**for covalent modification**			
*#Oligosaccharyltransferase (OST-A)*		ERM	
− RibophorinI (Rpn1)	1956		
− RibophorinII (Rpn2)	527		
− OST48	273		CDG
− Dad1	464		
− OST4			
− TMEM258			
− Stt3A *	430		CDG
− DC2			
− Kcp2			
*Oligosaccharyltransferase (OST-B)*			
− RibophorinI (Rpn1)	1956		
− RibophorinII (Rpn2)	527		
− OST48	273		CDG
− Dad1	464		
− OST4			
− TMEM258			
− Stt3B *	150		CDG
− TUSC3			CDG
− MagT1	33		
*Signal peptidase (SPC-A)*		ERM	
− SPC12	2733		
− SPC18 * (SEC11A)			
− SPC22/23	334		
− SPC25	94		
*Signal peptidase (SPC-C)*		ERM	
− SPC12	2733		
− SPC21 * (SEC11C)			
− SPC22/23	334		
− SPC25	94		
*GPI transamidase (GPI-T)*		ERM	
− GPAA1	9		
− PIG-K	38		
− PIG-S	86		
− PIG-T	20		
− PIG-U	42		

^1^ Abundance refers to the concentration (nM) of the respective protein in HeLa cells, as reported by Hein et al. [45]. ^2^ Localization refers to the functional intracellular localization(s) of the respective protein [1,2,3,4,35,36,41], i.e., C, Cytosol, ERL, ER lumen, ERM, ER membrane, PexM, and Peroxisome membrane. ^3^ Alternative protein names are given in parentheses. ^4^ Complexes are indicated by italics. Abbreviations for the protein names: EMC, ER membrane (protein) complex; GET, guided entry of tail-anchored proteins; SEC, (protein involved in) secretion; SND, SRP-independent; SR, SRP receptor; SRP, signal recognition particle; SSR, signal sequence receptor; TMEM, transmembrane (protein); TRAM, translocating chain-associating membrane (protein); TRAP, translocon-associated protein; TRC, transmembrane recognition complex. ^5^ Diabetes was linked to the particular protein in mice. ^6^ Abbreviation for diseases: CDG, congenital disorder of glycosylation; CVID, common variable immunodeficiency; GBM, glioblastoma multiforme; HUS, hemolytic-uremic syndrome; MSS, Marinesco-Sjögren syndrome; PLD, polycystic liver disease; TKD, tubulointerstitial kidney disease, as reported by Sicking et al. [34]. # indicates ribosome binding ability; ^§^ indicates ion channel activity; * indicates enzymatically active subunit.

**Table 2:** Statistics for the identification of putative PEX3 clients in comparison to the previously identified clients for ER membrane targeting and translocation components.

Proteins	PEX3	Z ^1^	RRBP1 ^2^	KTN1 ^2^	SEC61 ^2^	TRAP ^2^	CDG ^1,2^
Quantified proteins	8178	6328	4813	4947	7212	7670	5920
Statistically analyzed proteins	6488	6328	4813	4947	5129	5911	5920
representing the secretory pathway (%)	29	29	26	27	26	27	36
Proteins with SP (%)	7	7	6	6	6	7	nd ^3^
N-Glycoproteins (%)	9	9	8	8	8	8	nd
Membrane proteins (%)	13	13	12	13	12	13	nd
Positively affected proteins	0	97	157	25	342	77	39
Negatively affected proteins	13	141	141	45	482	180	279
representing the secretory pathway (%)	54	39	37	41	61	40	36
Negatively affected proteins with SP (%)	8	19	18	7	41	22	12
Negatively affected N-glycoproteins (%)	8	21	17	18	45	23	17
Negatively affected membrane proteins (%)	31	18	18	22	36	26	23
Negatively affected proteins with SP	1	27	21	3	197	38	34
including N-glycoproteins	0	23	16	3	158	28	30
corresponding to %	0	85	76	100	80	74	88
including membrane proteins	1	6	6	1	77	19	16
corresponding to %	100	22	29	33	39	50	53
Negatively affected proteins with TMH	3	16	18	8	98	22	41
including N-glycoproteins	1	6	7	4	56	11	17
corresponding to %	33	38	39	50	57	50	41
Negatively affected peroxisomal proteins	1	12	0	1	1	0	1
corresponding to %	8	9	nd	2	0	nd	0
including membrane proteins	1	6	nd	0	1	0	1
corresponding to %	100	50	nd	nd	100	nd	100
Negatively affected mitochondrial proteins	0	14	6	1	29	14	21
corresponding to %	nd	10	4	2	1	1	1
including membrane proteins	nd	4	3	0	11	3	8
corresponding to %	nd	29	50	nd	38	21	38

^1^ Z and CDG refer to immortalized fibroblasts from patients suffering from Zellweger syndrome or a congenital disorder of glycosylation. ^2^ Refers to siRNA-mediated knockdown HeLa cells, and was previously published [33,46]. ^3^ nd, not determined.

**Table 3:** Negatively affected proteins in PEX3-deficient cells, i.e., putative PEX3 substrates.

Gene	Subcellular Location	Membrane Protein Type	SS or TMH
ACBD5	Peroxisome membrane	Single-spanning membrane protein	
COLEC12	Membrane	Single-spanning type II membrane protein	TMH
LRRC15	Membrane	Single-spanning type I membrane protein	SP
PEX3	Peroxisome membrane	Single-spanning membrane protein	
TOR1AIP1	Nuclear envelope inner membrane	Single-spanning membrane protein	TMH
COL1A1	Secreted, Extracellular space, Extracellular matrix		SP
AGPS	Peroxisome membrane		
ACAD11	Peroxisome, Mitochondrion		
STX6	Golgi apparatus membrane	Tail-anchored membrane protein	tail anchor
CCDC136	Acrosome membrane, Secretory vesicle, Cytoplasmic vesicle	Tail-anchored membrane protein	tail anchor
FAR1	Peroxisome membrane	Tail-anchored membrane protein	tail anchor
PXMP2	Peroxisome membrane	Multi-spanning membrane protein	
ATL1	Cell projection, Golgi apparatus membrane, ER membrane,	Hairpin membrane protein with one HP	hairpin
COL6A2	Extracellular matrix, Membrane, Secreted, Extracellular space		SP
LOX	Extracellular space, Secreted		SP
ERMP1	ER membrane	Multi-spanning membrane protein	TMH
CYBRD1	Membrane	Multi-spanning membrane protein	TMH
TMUB2	Membrane	Multi-spanning membrane protein	TMH
ABCD3	Peroxisome membrane	Multi-spanning membrane protein	
SCP2	Peroxisome, Mitochondrion, Cytoplasm		
CDCP1	Secreted, Cell membrane		SP
COL6A3	Extracellular space, Secreted, Extracellular matrix		SP
TMEM237	Cell projection, Membrane, Cilium	Multi-spanning membrane protein	TMH
ENPP4	Cell membrane	Single-spanning type I membrane protein	SP
HTRA1	Cell membrane, Secreted, Cytoplasm, Cytosol		SP
VAMP3	Synapse, Membrane, Cell junction, Synaptosome	Tail-anchored membrane protein	tail anchor
MFGE8	Membrane, Secreted		SP
PRSS23	Secreted		SP
DHRS4	Peroxisome, Nucleus		
ITGB5	Membrane	Single-pass type I membrane protein	SP
FBLN1	Extracellular space, Secreted, Extracellular matrix		SP
COL6A1	Extracellular space, Secreted, Extracellular matrix		SP
PCSK9	Endosome, Golgi apparatus, Cell surface, Secreted, ER, Lysosome		SP
CTHRC1	Extracellular space, Secreted, Extracellular matrix		SP
DHRSX	Secreted		SP
HLA-C	Membrane	Single-spanning type I membrane protein	SP
CCDC80	Secreted, Extracellular space, Extracellular matrix		SP
RTN3	ER membrane, Golgi apparatus membrane	Hairpin membrane protein with two HP	hairpin
ENPP1	Secreted, Basolateral cell membrane, Cell membrane	Single-spanning type II membrane protein	TMH
PLOD2	Rough ER membrane		SP
RHOT1	Mitochondrion outer membrane		
COL11A1	Extracellular matrix, Extracellular space, Secreted		SP
NDUFV3	Mitochondrion inner membrane		
PCOLCE	Secreted		SP
AIFM2	Membrane, Mitochondrion outer membrane, Lipid droplet	Single-spanning membrane protein	TMH
MAN1A1	Golgi apparatus membrane	Single-spanning type II membrane protein	TMH
ACBD7	Cytosol		
ICAM1	Membrane	Single-spanning type I membrane protein	SP
CTSB	Lysosome, Melanosome, Secreted, Extracellular space		SP
DHRS7B	ER membrane	Single-spanning type II membrane protein	TMH
LAMA4	Extracellular matrix, Extracellular space, Secreted		SP
LEPREL1	ER, Golgi apparatus		SP
PEX13	Peroxisome membrane	Single-spanning membrane protein	
PDIA5	ER lumen		SP
CTHRC1	Extracellular space, Secreted, Extracellular matrix		
FKBP7	ER lumen		SP

The proteins are listed according to the decreasing negative effects of PEX3 depletion. The colors refer to peroxisomal proteins (yellow), mitochondrial proteins (brown), and proteins of the secretory pathway with SP, TMH, tail anchors (green) or hairpins (orange). As compared to Table S4, the GO annotation for TOR1AIP1, the hairpin of RTN3, and the definitions of the membrane protein types were taken from GeneCards (https://www.genecards.org; last accessed on 1 September 2021). In addition, the term “TMH” is used here only for proteins of the secretory pathway. Red letters refer to incomplete annotations (see text for details). HP, hairpin.

## Data Availability

The novel MS proteomics data were deposited to the ProteomeXchange Consortium via the PRIDE partner repository, with the dataset identifier PXD012005 (http://www.proteomexchange.org, accessed on 18 October 2021). In addition, all of the data are available from the authors.

## Supplementary Materials

The Supplementary Materials are available online at https://www.mdpi.com/article/10.3390/ijms222313028/s1.

## Abbreviations

ATL	Atlastin
CDG	Congenital disorder of glycosylation
EMC	ER membrane complex
ER	Endoplasmic reticulum
GET	Guided entry of tail-anchored proteins
GO	Gene ontology
GPI	Glycosylphosphatidylinositol
LD	Lipid droplet
PEX	Peroxin
RAMP	Ribosome-associated membrane protein
RNC	Ribosome-nascent chain complex
RTN	Reticulon
SEC	(Protein involved in) secretion
SND	SRP-independent
SP	Signal peptide
SR	SRP receptor
SRP	Signal recognition particle
SSR	Signal sequence receptor
TMEM	Transmembrane (protein)
TMH	Transmembrane helix
TRAM	translocating chain-associating membrane (protein)
TRAP	Translocon-associated protein
TRC	Transmembrane recognition complex
Z	Zellweger (patient fibroblasts)
