# Supplementary material for: Quantitative Proteomics and Differential Protein Abundance Analysis after the Depletion of PEX3 from Human Cells Identifies Additional Aspects of Protein Targeting to the ER
Source: Int J Mol Sci. 2021 Dec 1;22(23):13028. doi: 10.3390/ijms222313028 (PMC8658024; doi:10.3390/ijms222313028)
Supplement: Supplementary file 1 [file ijms-22-13028-s001.zip › Figure S1.pdf]

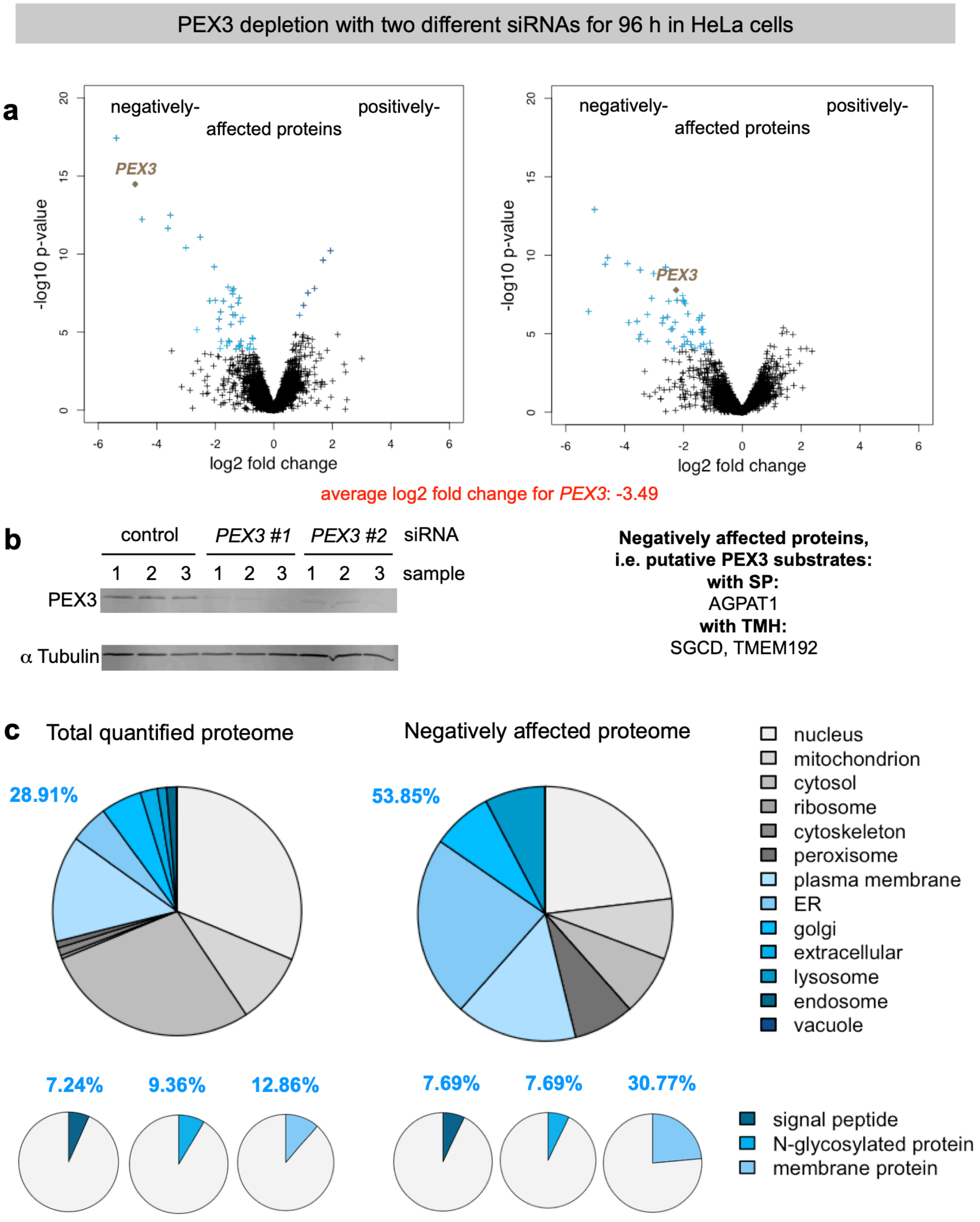

Figure S1. Volcano plots and Gene Ontology (GO) enrichment after PEX3 depletion in HeLa cells. (a) Differentially affected proteins were characterized by the mean difference of their intensities plotted against the respective permutation false discovery rate-adjusted *p*-values in volcano plots. The results for a single targeting siRNA are shown in each panel, PEX3 is highlighted. In addition, the proteins, which were negatively affected by both targeting siRNAs are given below the right panel. (b) Knock-down efficiencies were evaluated by western blot. Molecular mass values are indicated in kilodaltons (KDa). Only the area of interest of the blot is shown, the original images are shown in Supplementary Materials. (c) Classification of putative PEX3-clients was based on GO enrichment factors where the results from the complete set of quantified proteins in the left panel are compared with the negatively affected proteome. Protein annotations of SPs, membrane location, and N-glycosylation in humans were extracted from UniProtKB, and used to determine the enrichment of GO annotations among the negatively affected proteins.
