# Supplementary material for: Quantitative Proteomics and Differential Protein Abundance Analysis after the Depletion of PEX3 from Human Cells Identifies Additional Aspects of Protein Targeting to the ER
Source: Int J Mol Sci. 2021 Dec 1;22(23):13028. doi: 10.3390/ijms222313028 (PMC8658024; doi:10.3390/ijms222313028)
Supplement: Supplementary file 1 [file ijms-22-13028-s001.zip › MS blots PEX3.pdf]

Original blots, Figure 2
Molecular mass markers are indicated

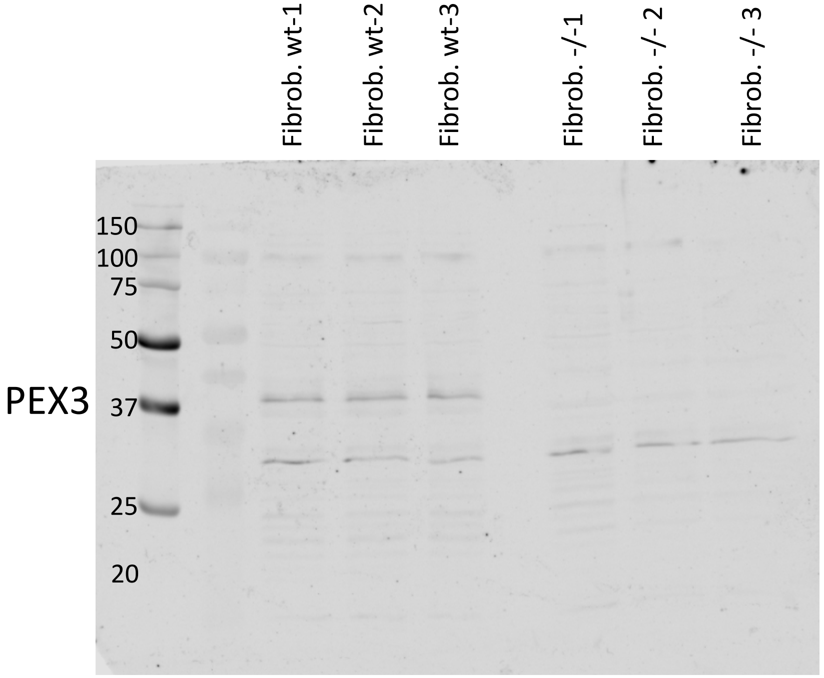

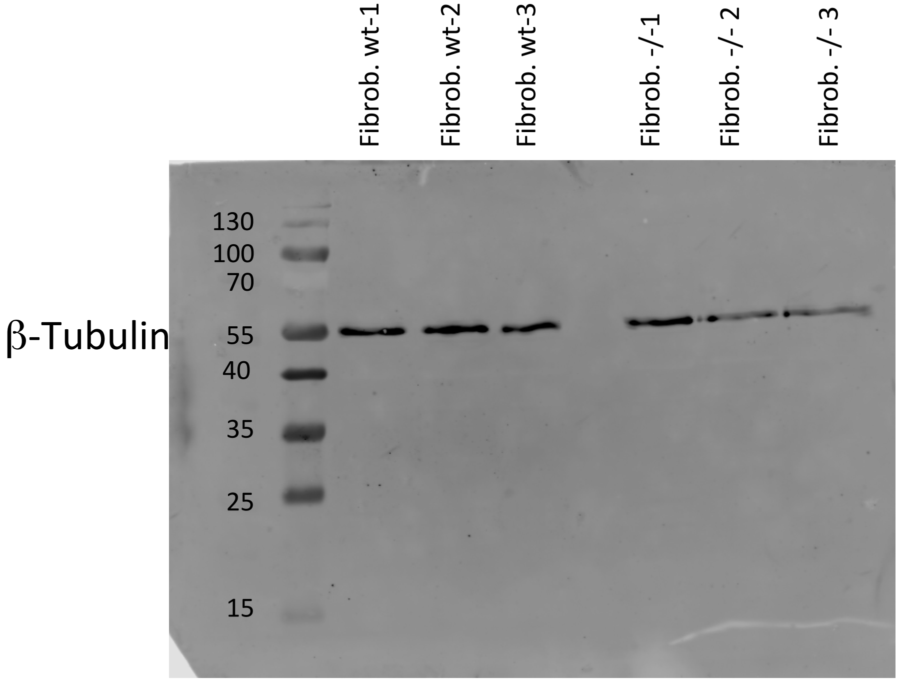

Original blots, Figure 3, a and b
Molecular mass markers are indicated

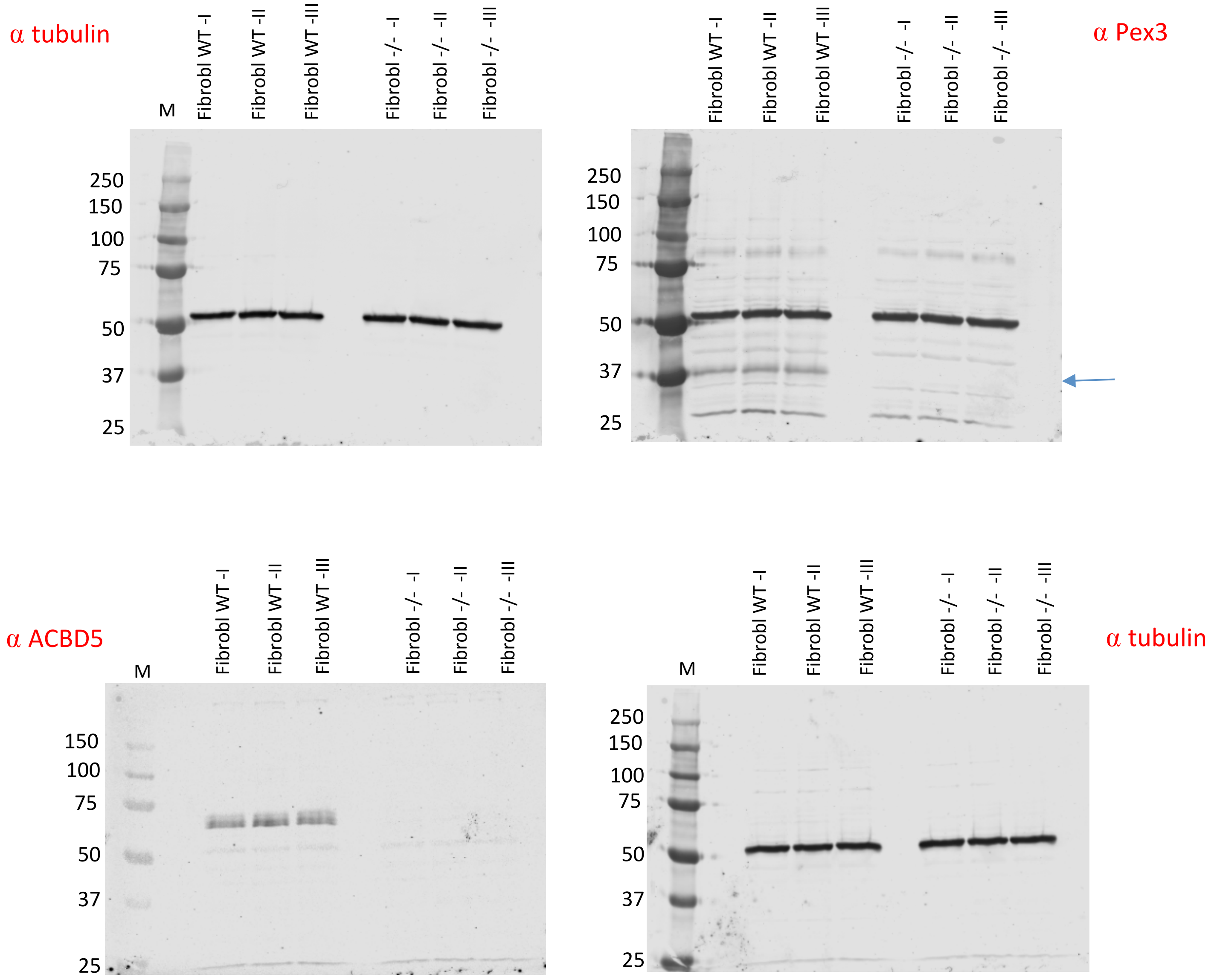

Original blots, Figure 3, c and d
Molecular mass markers are indicated

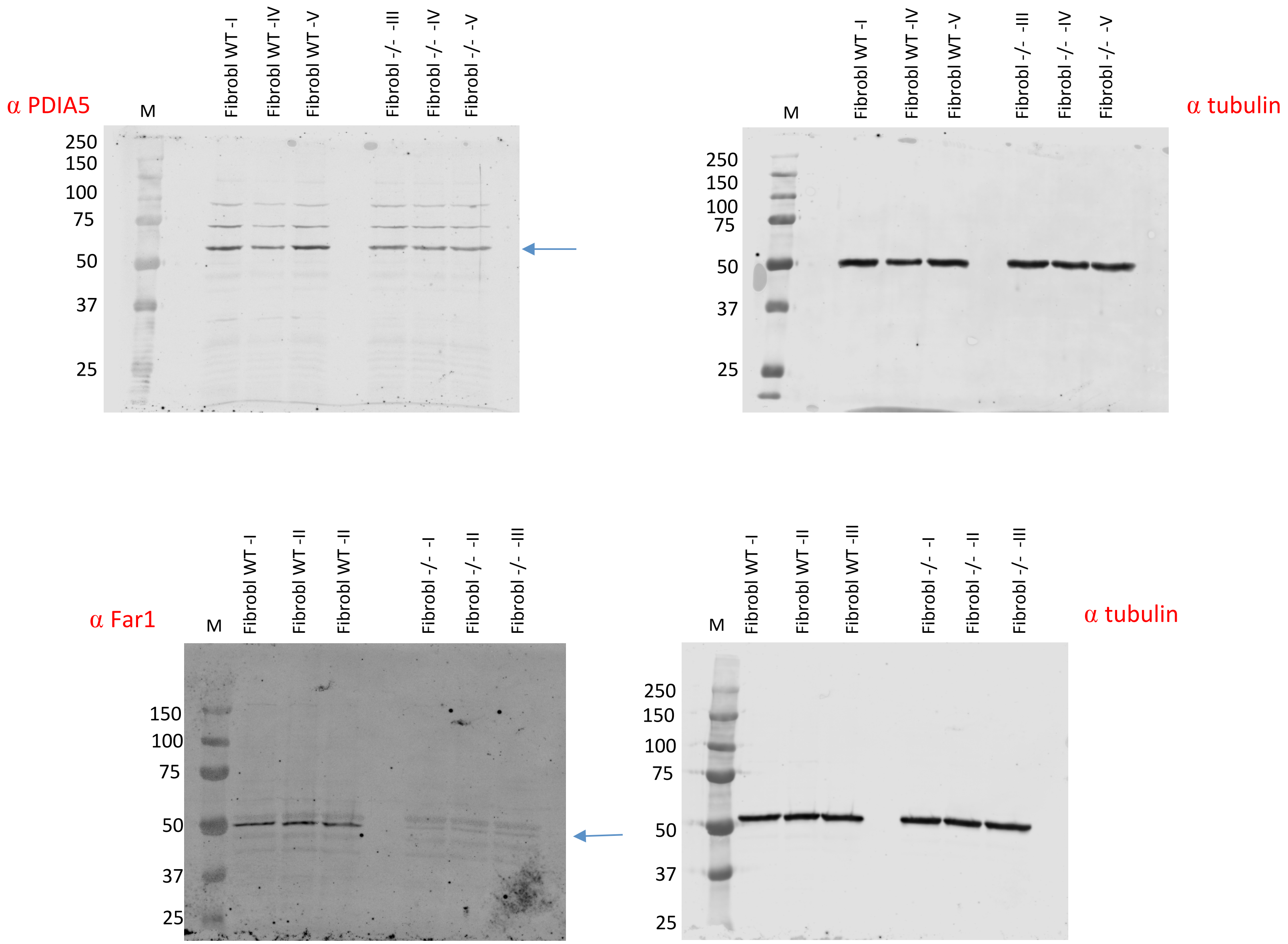

Original blots, Figure 3, e and f
Molecular mass markers are indicated

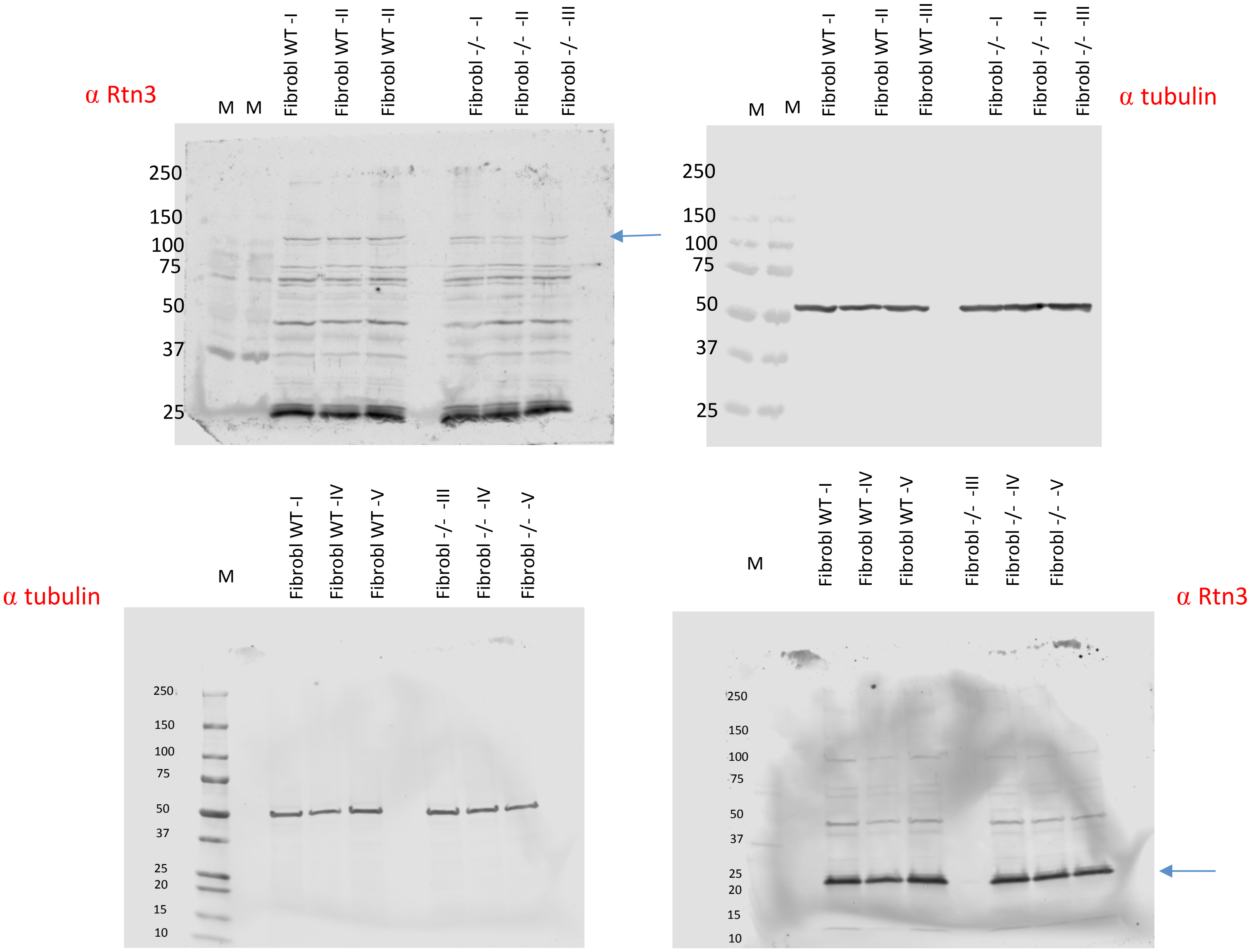

Original blots, Figure S1
Molecular mass markers are indicated

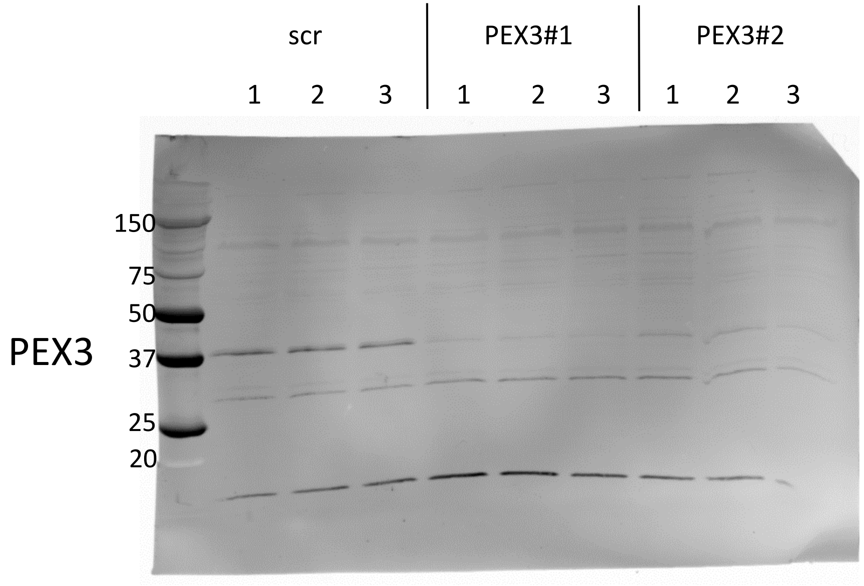

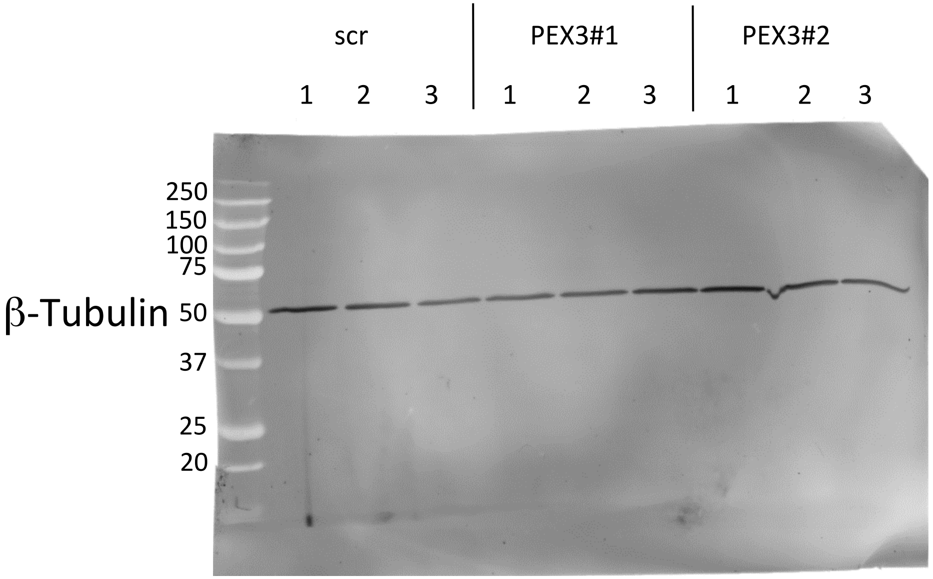

Silencing in HeLas
