# Supplementary material for: Quantitative Proteomics and Differential Protein Abundance Analysis after the Depletion of PEX3 from Human Cells Identifies Additional Aspects of Protein Targeting to the ER
Source: Int J Mol Sci. 2021 Dec 1;22(23):13028. doi: 10.3390/ijms222313028 (PMC8658024; doi:10.3390/ijms222313028)
Supplement: Supplementary file 1 [file ijms-22-13028-s001.zip › PEX3_Description of Supplementary Tables.pdf]

Description of Supplementary Tables

File Name: Table S1_PEX3_all.xlsx

Description: Complete list of genes corresponding to proteins quantified after PEX3 depletion in HeLa cells. Gene names, protein accession numbers (ID), log2 fold changes resulting from siRNA-mediated PEX3 depletion, and -log10 p values are indicated. Minus sign in front of fold change denotes negatively affected proteins. The number of listed proteins differs from the total number of quantified proteins because some proteins were quantified in less than two of the triplicates. The original Orbitrap data for all quantified proteins are deposited at Proteome Exchange: http://www.proteomexchange.org.

File Name: Table S2_PEX3_full_lo.xlsx
Description: Proteins that were negatively affected by PEX3 depletion in HeLa cells, i.e. putative PEX3 clients. Gene names, protein accession numbers, and log2 fold changes resulting from siRNA-mediated PEX3 depletion are presented together with full protein names and Gene Ontology (GO) annotations, presence of N-terminal signal peptide (SP) or N-terminal transmembrane helix (TMH), number of N-glycosylation sites (Glycosylation sites), amino acid sequences of SP or TMH (in single letter code), all as extracted from UniProtKB entries using custom scripts. Proteins are listed according to decreasing negative effects of PEX3 depletion.

File Name: Table S3_PEX3_Zellweger_all.xlsx
Description: Complete list of genes corresponding to proteins quantified in Zellweger patient fibroblasts with PEX3 deficiency. Gene names, protein accession numbers (ID), log2 fold changes resulting from PEX3 deficiency, and -log10 p values are indicated. Minus sign in front of fold change denotes negatively affected proteins. The original Orbitrap data for all quantified proteins are deposited at Proteome Exchange: http://www.proteomexchange.org.

File Name: Table S4_PEX3_Zellweger_full_lo.xlsx
Description: Proteins that were negatively affected by PEX3 deficiency in Zellweger fibroblasts, i.e. putative PEX3 clients. Gene names, protein accession numbers, and log2 fold changes resulting from PEX3 deficiency are presented together with full protein names and Gene Ontology (GO) annotations for subcellular location(s), presence of N-terminal signal peptide (SP) or N-terminal transmembrane helix (TMH), number of N-glycosylation sites (Glycosylation sites), amino acid sequences of SP or TMH (in single letter code), all as extracted from UniProtKB entries using custom scripts. Proteins are listed according to decreasing negative effects of PEX3 deficiency.

File Name: Table S5_PEX3_Zellweger_full_up.xlsx
Description: Proteins that were positively affected by PEX3 in Zellweger patient fibroblasts. Gene names, protein accession numbers, and log2 fold changes resulting from PEX3 deficiency are presented together with Gene Ontology (GO) annotations for subcellular location(s), presence of N-terminal signal peptide (SP) or N-terminal transmembrane helix (TMH), number of N-glycosylation sites (Glycosylation sites), amino acid sequences of SP or TMH (in single letter code), all as extracted from UniProtKB entries using custom scripts. Proteins are listed according to decreasing positive effects of PEX3 deficiency.

File Name: Table S6_HeLaZoverlap.docx
18 ut of 31 putative PEX3 putative clients in Zellweger patient fibroblasts were also negatively affected by PEX3 depletion in HeLa cells but did not meet the stringent significance threshold (in orange). The numbers were extracted from Table S1.

File Name: Table S7_proteomeXchange_identifiers.docx
The table gives the dataset identifiers for the novel datasets.
