# Supplementary material for: Quantitative Proteomics and Differential Protein Abundance Analysis after the Depletion of PEX3 from Human Cells Identifies Additional Aspects of Protein Targeting to the ER
Source: Int J Mol Sci. 2021 Dec 1;22(23):13028. doi: 10.3390/ijms222313028 (PMC8658024; doi:10.3390/ijms222313028)
Supplement: Supplementary file 1 [file ijms-22-13028-s001.zip › Table S7_proteomeXchange_identifiers.pdf]

The mass spectrometry proteomics data (.raw and .txt files) have been deposited to the ProteomeXchange Consortium via the PRIDE partner repository with the dataset identifiers: PXD012005 (http://www.proteomexchange.org).

Orbi2695	PEX3 experiment & Zellweger patient fibroblasts
Sample 1-3	scr control siRNA
Sample 4-6	PEX3 siRNA #1
Sample 7-9	PEX3 siRNA #2
Sample 10-12	control fibroblasts #3
Sample 13-15	Zellweger patient fibroblasts ΔPEX3
